# Gauging Gait Disorders with a Method Inspired by Motor Control Theories: A Pilot Study in Friedreich’s Ataxia

**DOI:** 10.3390/s21041144

**Published:** 2021-02-06

**Authors:** Arnaud Gouelle, Samantha Norman, Bryanna Sharot, Stephanie Salabarria, Sub Subramony, Manuela Corti

**Affiliations:** 1 Gait and Balance Academy, ProtoKinetics, Havertown, PA 19083, USA; 2 Laboratory Performance, Santé, Métrologie, Société (PSMS), UFR STAPS (University of Sport Sciences), 51100 Reims, France; 3 Department of Pediatrics, College of Medicine, University of Florida, Gainesville, FL 32610, USA; samantha.norman@peds.ufl.edu (S.N.); bsharot@peds.ufl.edu (B.S.); ssalabarria@peds.ufl.edu (S.S.); m.corti@peds.ufl.edu (M.C.); 4 Department of Neurology, College of Medicine, University of Florida, Gainesville, FL 32610, USA; s.subramony@neurology.ufl.edu

**Keywords:** organization, variability, walk ratio, gait scores, pressure walkway

## Abstract

To date, it has been challenging for clinicians and researchers alike to use the multiple outcome measures available to create a meaningful clinical picture and perform effective longitudinal follow-up. It has been found that instrumented gait analysis can provide information associated with a patient’s performance and help to remedy the shortcomings of the currently available outcome measures. The goal of this methodological article is to set the background and justify a new outcome measure inspired by the motor control theories to analyze gait using spatiotemporal parameters. The method is applied in a population of individuals living with Friedreich’s ataxia (FRDA), a neurodegenerative disease. The sample population consisted of 19 subjects, 11 to 65 years of age with FRDA, who either ambulated independently, with a cane, or with a rollator. Three scores based on the distance from healthy normative data were used: Organization Score, Variability Score, and an overall measurement, the Global Ambulation Score. The scores were then compared to the Scale for Assessment and Rating of Ataxia (SARA) Gait Score (SARA-GS), a clinical scale currently being used for gait analysis in FRDA. Organization Scores demonstrated a longitudinal deterioration in the gait characteristics from independent ambulators to those who ambulated with a rollator. Variability Scores mostly reflected dynamic instability, which became greater as the requirement of an ambulation aid or the switch from a cane to a rollator was imminent. The global value given by the Global Ambulation Score, which takes into consideration both the Organization Score, the Variability Score, and the level of assistive device, demonstrated a logarithmic relationship with the SARA-GS. Overall, these results highlight that both components introduced should be analyzed concurrently and suggest that the Global Ambulation Score may be a valuable outcome measure for longitudinal disease progression.

## 1. Background

### 1.1. Motivation

There are two objectives underlying the entirety of clinical research on diseases that affect gait and dynamic balance abilities. The first objective involves identifying gait characteristics that differentiate between individuals with a specific disease and healthy controls. This objective requires a more comprehensive understanding of the underlying mechanisms of the disorders itself hence, subtle gait impairments present in the prodromal stage likely reflect early degeneration and may be used as a diagnostic preclinical screening tool [1] for neurodegenerative diseases. As with all diseases, earlier identification can lead to earlier intervention when available. The second objective involves longitudinal studies of gait parameters critical to analyzing a disease’s natural history and improving prognosis accuracy. This knowledge is essential to proving the adequacy and sensitivity to change [2,3], so these outcome measures could serve as translational clinical endpoints for future clinical trials in this disease model. To achieve these two objectives, spatiotemporal parameters have been extensively used. Recent studies highlight that reporting changes in individual gait parameters (e.g., gait speed or step length) do not capture the full complexity of gait given the interdependency of these variables [4]. Factor analysis allows for the exploration of the covariance between individual gait parameters. This analysis provides the advantage of limiting gait parameters to a few gait domains (most often referred to as pace, rhythm, variability, asymmetry, postural control, and phases) that provide the clinician with an idea of which gait parameters to monitor [5,6]. However, the implementation of exploratory factor analysis in rare diseases is challenging due to the smaller sample sizes, as the analysis requires three to 20 times the number of variables. Therefore, smaller sample sizes must be treated with caution [7,8]. Nonetheless, it is often difficult to clinically determine which changes reflect disturbances, or which are compensatory mechanisms to avoid loss of balance, falls, and subsequent injury. Interpreted altogether, all are largely important in developing an effective and individually curated therapeutic approach for each patient.

Thus, the goal of this paper is to present a new approach for understanding gait mechanisms inspired by motor control theories and to provide a simplified scoring system for gait assessment. Additionally, following analysis of the gait data captured, we illustrate the strength of this method for clinical evaluation and follow-up of walking ability in patients with Friedreich’s ataxia.

Friedreich’s ataxia (FRDA) is the most common of inherited ataxias affecting one in 20,000 to one in 50,000 people in Caucasian populations of Europe, the Middle East, South Asia, and North Africa [9]. FRDA typically begins in childhood and adolescence causing progressive incoordination, sensory loss, and muscle weakness. In time, this leads to an ataxic gait and gradual loss of ambulation. However, there are sizeable disparities in motor performance and symptom manifestation from one patient to another, given the genetic origin of the disease. FRDA is caused by autosomal recessive GAA trinucleotide expansions in the first intron of the FXN gene on the proximal long arm of chromosome 9, which interferes with frataxin transcription [9]. Patients with FRDA are homozygous for massive expansions of a GAA triplet-repeat sequence [10,11]. The length of triplet repeat appears to influence the clinical phenotype, time of onset, symptom severity, disease progression, and is directly correlated with expansion size [9,11,12]. Symptoms of FRDA typically become evident around puberty [13], however, the age of onset may vary substantially, even within a sibship. Early-onset may be around the age of two years, meanwhile, late-onset FRDA is defined as having onset after 25 years of age [9]. Occasionally, patients become symptomatic only in their sixth or seventh decade of life [14,15]. Clinical features of FRDA combine cerebellar, pyramidal syndromes, and axonal neuropathy, which cause coordination deficits, loss of proprioception, and balance difficulties. Symptoms and deficits vary highly from one patient to another with gait parameters not following a typical linear progression [4]. Additional orthopedic features like scoliosis [16] or foot deformities (e.g., pes cavus, equinovarus, or toe claw) [17,18] are seen in more than two-thirds of patients and contribute to the differences between individuals. Lastly, as the disease progresses and gait worsens, assistive devices such as canes, crutches, walkers, or rollators become necessary to facilitate safe, upright locomotion, maintain mobility, and prevent the deleterious effects of immobility [19].

Following our observations, we believe our approach could contribute to a better understanding of the mechanisms underlying the loss of ambulation in individuals with FRDA. We also aim to identify sensitive and objective parameters to quantify changes over time in patients with different disease severity levels.

### 1.2. Motor Control: From Theoretical Considerations to Implication for Gait Assessment

Motor control theories attempt to explain how a movement is produced and regulated. Historically, most of the work and considerations about motor control have been based on the notions of motor program [20]. In this concept, motor control implies that the individual develops a number of patterns and creates rules for the parametrization of each movement (angles, velocity, stiffness, etc.) with some fluidity allowing for edits to be made to the movement when necessary. All movement is carried out by the central nervous system—the body’s control center—whose role is to integrate the information gathered from the environment and communicate an action plan to the body’s motor system. However, no biological system can be precise and free from external disturbances, so error correction must take place. A retroactive control is required through regulation loops, of proprioceptive, vestibular, and visual origin, to adapt the various walking parameters to the constraints of the external environment. In this theoretical framework, the measurements observed as motor output are the sum of the signal and the undesired disturbances within the signal (i.e., noise). Therefore, the invariance of the movement is interpreted as the natural order of the biological system and the variability reflects the physiological inability to produce and regulate a movement. Clinically, gait variability is often interpreted as noise, which must be removed, disregarding its potential for regulation. On the other hand, if a patient must deal with different or additional constraints (e.g., pathology), it becomes necessary to re-optimize the processes of motor control. The central nervous system would have to integrate new constraints, even a new motor program. In this perspective, it is unlikely, within the initial stages of a modified motor scheme’s implementation, that the scheme will effectively ensure stability as the patient transitions from older to more novel motor processes.

**Figure 1:**
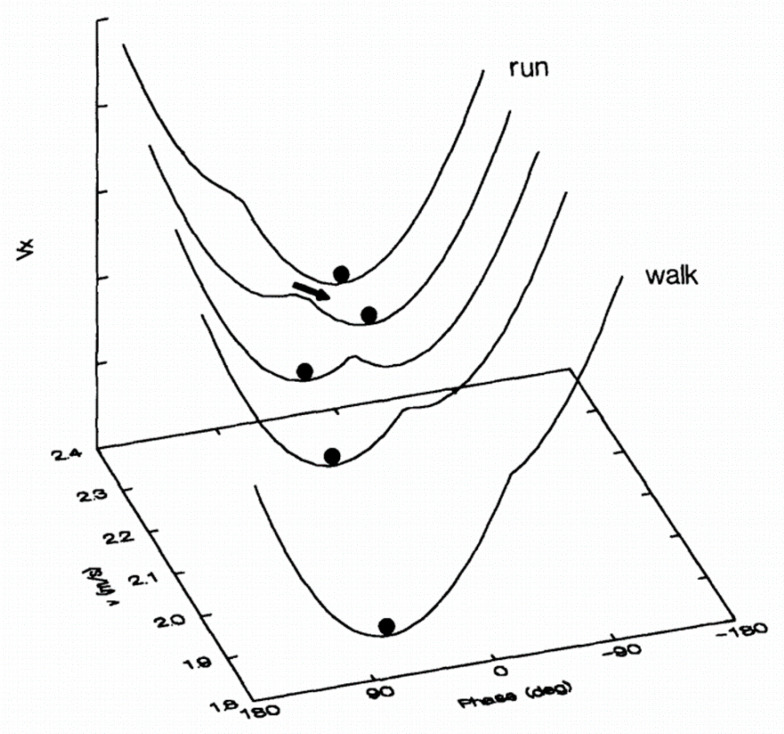
Schematic of a potential function V(x) for the walk–run transition (from Diedrich and Warren, 1995). As speed (y) increases, the system (represented by the ball) moves from a walking attractor with a stable relative phase (x) and preferred speed through an unstable region and jumps to a running attractor with a different stable relative phase and preferred speed. The reverse run-walk jump occurs at a lower speed (hysteresis).

Another approach of motor control allows for further self-organization. Outside periods when a direct cognitive intervention is required to take control of the movement (e.g., turning towards the right at next street corner), the dynamic systems theory [21,22] postulates that the behavior (e.g., walking pattern) observed in a complex system (e.g., the human body) emerges spontaneously from constraints related either to the task (e.g., adding a cognitive task in a dual-task paradigm), the organism (e.g., pathological limitations), and/or the environment (e.g., walking within a crowd). Under standardized conditions, a patient’s gait pattern should represent the visible translation of self-organization with emergent, spontaneous coordination among the multiple possibilities, while utilizing minimum energy expenditure. The main trait of this coordination, known as an attractor, is the stability of the corresponding behavior [22]. Stability refers to the coordinative pattern’s resistance to change in response to a perturbation measured by variance, deviation from the attractor state, or the ability to return to an attracter state rapidly [23]. Repulsive states are also possible but cannot be maintained because stability is close to zero and any slight disturbance will cause a phase change in the system. The most illustrative example for locomotion (Figure 1) is the walk–run transition [24]. When placed on a treadmill at 5 km/h, a person spontaneously starts walking. Likewise, at 10 km/h, the person spontaneously begins to run. In these situations, walking and running represent two high stability attractors. Now, if the walking speed increases (or the running speed decreases), a transition from walk to run (or from run to walk) will occur around 7.5 km/h [24]. The transition implies the competition between the two attractors, resulting in unstable coordination around this speed, i.e., the subject can switch back and forth very quickly from one state to another, and increase the variability of stride duration [25]. In this perspective, preferred spontaneous gait is characterized by stable organization and minimal energy expenditure, while transitions between gait coordination are characterized by instability and increased energetic costs. Self-organization, which aims to respond to the constraints as much as possible, can therefore evolve if the latter changes.

The other significant contribution of the dynamic systems theory is the role given to variability. Contrary to the conventional cognitive approach, the presence of noise is essential in system dynamics. Variability is neither random nor meaningless, it has a structure which attests to its importance in the processes of motor control and regulation. It is not only unnecessary and harmful noise, but a noise that participates in an adaptive function [26] and has the potential to be a source of information on the different states available. Regarding human ambulation, multiple studies have demonstrated that the complex fluctuations in stride intervals, stride speeds, and stride lengths exhibit fractal patterns with inverse power-law memory [27,28]; that is, a change occurring at a given gait cycle can potentially influence another cycle dozens of steps later [29]. For clinics, the main interest of gait dynamics lies in providing information about whether a gait is highly controlled or more automated [29]. Therefore, it opens the door to understanding what is due to perturbation (primary disease) or regulation (adaptative processes). This strategy is especially useful when some changes seen in the gait characteristics emulate a higher risk for fall [30] and/or the adoption of a biomechanically safer, less destabilizing gait pattern [31]. However, as “long-term dynamics” suggests, these techniques require time-series captured along several minutes of walk to be reliable and caution must be used when assessing the fractal dynamics of gait variability over a period of 3 min [32]. Per extension, methods like Detrended Fluctuations Analysis cannot be used to study gait in people who are unable to perform 500 strides continuously [32].

On the other hand, the fluctuations identify a system’s degree of stability, e.g., its ability to maintain preferential coordination despite the disturbances. The lower the critical threshold of fluctuations, the less stable the coordination will be. Fluctuations explain the impossibility of maintaining a repulsive state, furthermore, showing that coordination can be stable despite a remarkably high level of fluctuations. An individual who falls due to a minor perturbation could be categorized as unstable. However, as van Emmerik et al. [33] wrote, “it is more difficult to determine the degree of stability when a system displays sufficient resilience to the perturbation. That is, how close is the system to shifting into an unstable state?”.

All these considerations lead us to look at three features:

a. The Organization of the Gait Pattern

Gait characteristics that differ significantly from control norms do not necessarily imply higher instability due to the self-organized nature of the movement. Again, it does not mean that this organization will require less energy expenditure or demonstrate less variability than a healthy gait, but it will be close to an optimum considering the patient’s constraints. However, this organization, which signifies a biomechanical adaptation, tends also to fix the degrees of freedom to a more manageable quantity to accomplish the walk [34]. Then, the residual capacity of adaptation is more limited. If there are mobilizable and adequate degrees of freedom for the task, the motor equivalence allows the person to adapt. However, if the instability increases, the overall amount of additional internal or external perturbations will become more complex to manage, until they are unmanageable.

b. The Amount of Variability

Two different gait patterns can still exhibit equivalent variability, while two similar gait patterns do not necessarily duplicate variability. Knowing how variable the person’s gait is provides insights about the stability of their gait pattern and could help define when to transition to using an assistive device. As mentioned above, when all adaptation capabilities are in play, the variability will inevitably increase, and an assistive device will become necessary. With limitations in gathering long-term FRDA patient datasets, it is not possible to build on nonlinear analysis methods to define when variability reflects perturbation or regulation. However, if regulation is seen, it is typically an answer to a perturbation. Therefore, the global amount of variability should be representative of both regulation and perturbation, at quite a similar level.

c. The Use and Type of Assistive Device

Ambulating without aids, with a cane, or with a walker/rollator should be thought of as three different coordination states, with a specific organization and critical threshold for stability for each state. From ambulating unassisted to ambulating with a cane, and from ambulating with a cane to ambulating with a walker/rollator, the base of support changes and the projection of the center of mass moves further. On the other hand, the degrees of freedom are increasingly restricted by the aids, resulting in a more constrained movement pattern. Just as we observe when a child learns to walk [35] or when they start acquiring a complex motor skill [36], more stiffness will be beneficial in avoiding potentially destabilizing motor patterns, yet leaves less adaptability to the system.

### 1.3. Quantifying Organization and Variability

Spontaneous ambulation speed is an established measure for functional capabilities and often used as an indication of performance and to monitor the level of progression. Gait speed was even suggested as the sixth vital sign of health [37], claiming it is one of the most important indications of the body’s functional status. However, speed is the product of step length and cadence. It is possible to produce the same speed by multiple configurations ranging from quick, small steps to slow, long steps [4].

As already demonstrated by Cavagna and Kaneko [38], preferential gait speed in a healthy individual is close to one that minimizes the energy expenditure per unit distance. However, that is true if the step rate is freely chosen. At the same speed, a forced step rate will increase the oxygen consumption required for the “free” step rate [39]. Interestingly, several studies by Sekiya further demonstrated that the most optimal energy consumption per distance is governed by a constant ratio between step length and step rate [40]. This “walk ratio” represents the relationship between the amplitude and the frequency of movement of the legs. It is calculated by the mean step length divided by the cadence. In control adults, the walk ratio is relatively invariant within a speed range, from extremely slow to extremely fast, independent of the gait speed [40]. An invariant walk ratio suggests that when healthy individuals ambulate faster or slower than their preferential speed, the relative proportion of step length and cadence in producing the gait speed is maintained. Thus, to move faster, there is an equivalent relative increase in step length and cadence.

**Figure 2:**
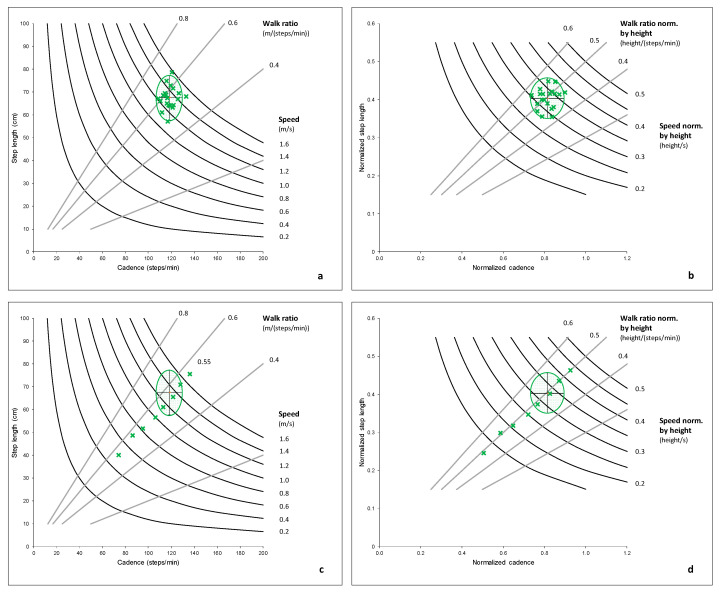
Representation of values for walking speed and walk ratio based on various step length—cadence configuration. (**a**,**b**) shows gait parameters from the healthy control group used to reference the developed scoring system (see Section 2.1). (**c**,**d**) displays data from one healthy adult who walked several paces from very slow to very fast. The green ellipse in the graphs demonstrates the 2-SD (standard deviation) range in the control group for step length and cadence.

Figure 2a,b shows the walk ratio and walking speed in the control group. The walk ratio is computed from the non-normalized step length and cadence shown in Figure 2a. Figure 2b presents a normalized walk ratio computed from step length, and cadence normalized by height [41]. Figure 2c,d displays data from a single, healthy adult who walked at several paces from extremely slow to extremely fast for an illustrative perspective. While the speed increased linearly through the condition, the walk ratio did not vary and stayed between 0.53 and 0.56 for the non-normalized values and between 0.48 and 0.51 for the normalized values. For an animation of virtual humans walking at the same speed but with different walk ratios, we advise watching the video provided as supplemental material in the work by Niay et al. [42].

Walking with a constant walk ratio would be optimal in terms of energy expenditure [43], temporal variability [44], spatial variability [45], and attentional demand [46]. Increased gait variability is evident when healthy controls walk slower or faster than their preferential speed, and when walking at their preferential speed with imposed step rates [45]. Recent work investigated the independent influence of gait speed and step length on stability and fall risk by decoupling each parameter from one another [47]. When healthy adults walked at their preferential speed but were asked to modify their step length, each one-standard-deviation (SD) increase in step length conferred a six times higher risk of falling. When they walked slower while maintaining a step length similar to that of their preferential gait, each one-SD decrease in gait speed increased their odds of falling by four. Lasty, a simultaneous change in speed and step length revealed that although slow gait leads to instability and falls, this is offset by the related decrease in step length. These results demonstrate that gait speed is important; however, the relative portion of step length and cadence, given by the walk ratio—what Espy et al. [47] were implicitly modulating—is key.

It appears that the combination of gait speed and walk ratio gives a detailed picture of the individual’s ambulation pattern, with information about the gait speed and how this is achieved in terms of spatial (step length) and temporal (cadence) organization. For this reason, we chose to develop a weighted composite score from the mean value of gait speed and walk ratio as both an organization score and a weighted composite score from their coefficient of variation as a variability score.

Below introduces an original scoring system that reflects both the organization and the variability of gait pattern, while also considering the use of an assistive device. We investigate how these outcomes relate to a clinical scoring of gait ability in FRDA. Then, we will provide an overview of the potential of the method to understand the longitudinal changes in gait and outline what happens when a patient is in a functional transition period, i.e., transitioning from no device to an assistive device or switching to a new device that offers greater support. As the neurodegenerative nature of FRDA amplifies instability, patients require adaptation to shifting functional constraints. We hypothesized that the organization score would worsen with the disease progression. Moreover, the variability score would reflect the level of instability within one coordination (e.g., without aid, with a cane, or with a rollator).

## 2. Materials and Methods

### 2.1. Participants

All FRDA subjects were participants of the University of Florida’s clinical trial titled Biomarkers in Friedreich’s Ataxia. Nineteen participants with FRDA, 11 to 65 years old (only one older than 49 years) were included. Demographics and clinical information are available in Table 1. To be eligible, participants were required to provide a confirmed genetic diagnosis of Friedreich’s ataxia and be between the ages of 8 and 70 (inclusive). Exclusionary criteria included pregnancy, unstable heart disease, heart transplant, or any other concurrent medical condition, that in the opinion of the principal investigator would make the subject unsuitable for participation. All subjects gave their informed consent for inclusion before they participated in the study. The study was conducted in accordance with the Declaration of Helsinki, and the protocol was approved by the Institutional Review Board of the University of Florida and declared on ClinicalTrials.gov (accessed on 6 February 2021) (NCT02497534).

Twenty asymptomatic, healthy adults from 18 to 41 years old (27 ± 7), without any other neurological conditions, gait disorders, or history of lower limb surgery, were included to derive an optimal gait reference for computation.

### 2.2. Procedures

Participants with FRDA were enrolled and followed on a continuous basis, every six months, between April 2018 and February 2020. They were examined by movement disorder specialists and graded on the Scale for Assessment and Rating of Ataxia (SARA) [48] and the modified Friedreich’s Ataxia Rating Scale (mFARS) [49,50]. The SARA has eight categories with an overall score ranging from 0 (no ataxia) to 40 (most severe ataxia). It includes a gait score, ranging from 0 (a normative walk, turning, and tandem walk) to 8 for the inability to ambulate, even supported. The mFARS is an adaptation of the FARS, a rating scale that was developed to quantitatively assess the severity of the neurologic features of FRDA. The assessments used in the mFARS relate to activities of daily living, such as standing, ambulating, pointing, and speaking. The scale consists of 4 sections that focus on specific areas of the body: bulbar function, upper limb coordination, lower limb coordination, and upright stability. It is utilized to measure change at single points in time over time.

Gait data collection utilized the Zeno Walkway (6.10 m × 0.61 m; ProtoKinetics, Havertown, PA 19083, USA). Researchers asked participants to balance and initiate gait once an automatic light turned on, after ten seconds, signaling them to ambulate. Once gait was initiated, participants completed three passes on the Zeno Walkway, turning off the mat after each pass. Evaluators instructed participants to walk at a speed they felt comfortable and safe doing. Once the walk was captured, trials were processed with the PKMAS software (ProtoKinetics, Havertown, PA 19083, USA).

Gait data collection occurred twice a year over two years, if participants remained ambulatory, with or without an assistive device (cane or rollator). Each participant completed one to four-time points depending on the enrollment date. Therefore, for this article, multiple time-points were only used to illustrate changes observed during the switch of ambulatory aids.

### 2.3. Computations

Three gait parameters were exported: velocity (m/s), step length (m), and step time (s). Step cadence (steps/min) was computed from the step time. These parameters have all demonstrated high reliability (ICC > 0.90) during test–retest in an heterogenous sample of patients with FRDA [51]. Before going further, velocity, step length and cadence were normalized to control for differences due to growth/stature. Adimensional parameters were calculated considering the participant’s height on the day of assessment and acceleration (g) relating to gravity [4,41], such that:

- Normalized Step length λ = step length/height
- Normalized Cadence φ = (cadence/60)/$\sqrt{g/height}$
- Normalized Velocity = velocity/$\sqrt{g\times height}$ = λ × φ
- Normalized Walk Ratio = λ/φ.

Step length and height were measured in meters, cadence in steps/minute, and velocity in meters/second.

The mean and coefficient of variation (CV) for the normalized velocity and normalized walk ratio were then computed. The coefficient of variation is the standard deviation of the gait parameter expressed as a percentage of the mean and explores the variability over the task. The computed mean and standard deviation of the control group for these parameters were used as a reference for the computation of z-scores. A z-score represents the number of SDs separating the parameter of an individual from the reference group’s mean value. Therefore, four parameters were obtained for each person in both the control and the FRDA groups: z-score for the mean normalized velocity ($zVn_{mean}$), z-score for the normalized walk ratio ($zWRn_{mean}$), z-score for the CV of normalized velocity ($zVn_{CV}$), z-score for the CV of normalized walk ratio ($zWRn_{CV}$).

Finally, by considering odd factors found by Espy et al. [47], i.e., one-SD increase in step length conferred six times higher odds of falling, while one-SD decrease in gait speed increased it by four, we computed the Organization and Variability Scores as:

- Organization Score (OrgS)
  = IF (zVn<0; $-\sqrt{\left(4\;{\left(zVn_{mean}\right)}^{2}+6\;{\left(zWRn_{mean}\right)}^{2}\right)}\;$; $\sqrt{\left(4\;{\left(zVn_{CV}\right)}^{2}+6\;{\left(zWRn_{CV}\right)}^{2}\right)}\;$)
- Variability Score (VarS)
  = $\sqrt{\left(4\;{\left(zVn_{CV}\right)}^{2}+6\;{\left(zWRn_{CV}\right)}^{2}\right)}$

A minus sign is introduced for the Organization Score when the z-score for the individual’s normalized velocity is negative, indicating a lower speed than the control reference group. This distinction will have a major significance in quickly differentiating individuals who walk faster than the norm.

A last score was finally introduced, the Global Ambulation Score, which aims to give a single global value and is defined as:

- Global Ambulation Score (GAS)
  = (abs(Organization Score)+abs(Variability Score))×Coefficient for Walking Aids

With Coefficient for Walking Aids being: 1 for unassisted gait; 2 for one cane; 3 for two canes or crutches (not represented in our current data); 4 for a rollator.

These coefficients were selected to follow the increase in support provided by the assistive device, from unilateral support given by one cane, to the bilateral support given by a rollator. Many classifications or questionnaires for the patient’s functional status look at an increasing need of assistance and/or walking aid in such a progressivity, with unassisted gait, unilateral or bilateral support. For example, the Functional Staging for Ataxia [49] includes stages from 0 to 6, with stage 3 indicating that the subject requires regular or periodic holding onto wall/furniture or use of a cane for stability and walking, and stage 4 indicates a subject who requires a walker, crutches, or two canes.

### 2.4. Graphical Representation

The most important aspect of the proposed method is to better understand the patient’s gait organization and its associated variability. Numbers are useful for follow-up quantification and statistical purposes, but we want to encourage clinicians to go beyond the values and understand what they mean. For this, graphical representation is best.

**Figure 3:**
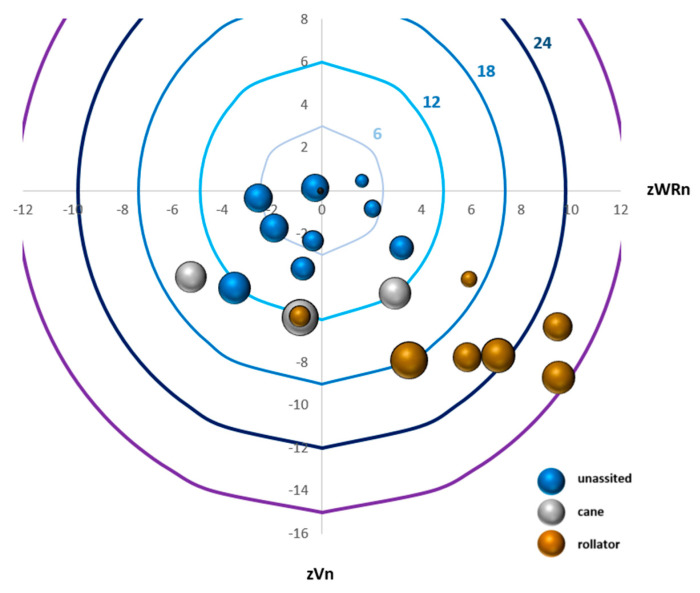
Graphical representation of the Organization Score and the Variability Score for the 19 patients with FRDA at baseline. Z-scores from healthy control for the normalized velocity (Y-axis: zVn) are first displayed in the function of the z-score for the normalized walk ratio (X-axis: zWRn). Circular curves represent the value for the Organization Score. Each assessment is shown as a 3D bubble chart with the area of the bubble reflecting the Variability Score. Finally, the color of the bubble illustrates the type of assistive device utilized: blue for unassisted walkers; grey for cane users; bronze for those who required a rollator. The black bubble at (0,0) is for the normative population reference.

Figure 3 provides direct visualization of the OrgS and how it is attained from velocity and walk ratio z-scores, the VarS, and the use of an assistive device.

We first graphed the z WRn mean as the x-axis and z Vn mean as the y-axis. Accordingly, the (0,0) of the graphic represents the control group’s mean as a reference. For the y-axis, a slower speed will be displayed under zero and a faster speed above. For the x-axis, a smaller walk ratio will be displayed on the left and a higher walk ratio on the right. Then, circular curves were added, which represent the OrgS. Each assessment is shown as a 3D bubble with the area of the bubble reflecting the VarS. Finally, by adding color to the bubble reflecting which assistive device was utilized by the individual, we obtain a full graphical representation.

### 2.5. Analysis Plan

Baseline assessments were used to examine the OrgS, VarS, and GAS in relation to the SARA Gait Score (SARA-GS). An independent Mann–Whitney U-test was operated to check for any differences (*p* < 0.05) between the participants who were unassisted and those who utilized a rollator. The degree of correlation between the scores measured and the SARA-GS assessed during the study visit was explored through linear regression models. Spearman rho coefficients were used as the correlation values.

As part of a preliminary analysis, we looked at subjects with multiple assessments who switched from one cane to a rollator over six months to illustrate how the changes in OrgS and GAS can help support and guide clinical decision-making.

## 3. Results

At baseline, nine participants ambulated without aids, three with a cane, and seven with a rollator. Due to the limited sample of cane users, statistical analysis was run only between unassisted walkers and rollator users. Based on the demographics and data collected, participants who utilized a rollator were older than the unassisted walkers (95% Confidence Interval, CI95%: 18.4–49.5 vs. 11.9–16.8 years-old). Additionally, those utilizing a rollator had lived with FRDA for the longest time (CI95%: 9.8–25.9 vs. 4.9–9.6 years) and had worse SARA-GS (3.0 ± 0.7 vs. 6.1 ± 0.7).

Figure 3 visually highlights the different levels of organization and variability through all participants. Table 1 provides mean, standard deviation, and CI95% for the parameters and the scores computed in this study, as well as significant differences between the group of independent ambulators and the group of individuals who used a rollator. The mean position of the control group had a 0 z-score and an SD of 1 for both the mean and coefficient of variation for speed and walk ratio. The CI95% for OrgS, VarS and GAS were [−1.8, 1.1], [1.8, 3.4] and [4.3, 6.5], respectively, and the minimum to maximum ranges were [−5.2, 4.3], [0.9, 8.1] and [2.0, 11, 9], respectively. In the FRDA participants, normalized walking speed was decreased with a rollator (0.12 ± 0.05 vs. 0.28 ± 0.05) and the normalized walk ratio was increased (0.71 ± 0.13 vs. 0.49 ± 0.08), leading to a higher negative OrgS than for the independent ambulators (−5 ± 5 vs. −21 ± 6). If a higher VarS was seen in the rollator group, there was no significant difference between assisted and unassisted individuals. The GAS, which considers a different factor according to the walking aid, demonstrated a higher range of values in the rollator group (42 ± 18 vs. 285 ± 107).

**Figure 4:**
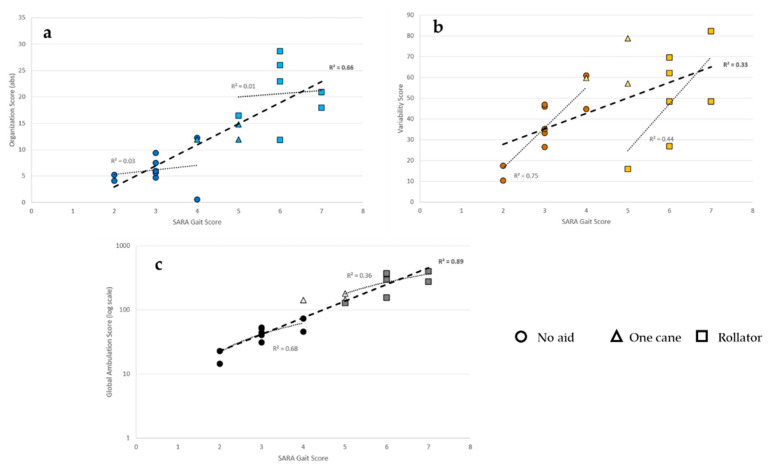
Organization Score (**a**), Variability Score (**b**), and Global Ambulation Score (**c**), logarithm scale) in the function of the SARA Gait Score. Linear regressions are modelized in each group of patients according to the need and type of assistive device and in all patients. The coefficient of determination (R²) of the Spearman rho coefficients is given to describe how much the regression matches the values’ distribution.

Regression models (Figure 4) showed a positive relationship between the OrgS (absolute) and the SARA-GS (ρ² = 0.66) through all the participants, however, this was not seen among subjects in the same group (i.e., unassisted or assisted). A moderate relationship was found between the VarS and the SARA-GS (ρ² = 0.33), but the relationship was stronger within the group of individuals ambulating independently (ρ² = 0.75) and the group of individuals ambulating with a rollator (ρ² = 0.44). The mathematical model explains the distribution of scores in each group rather than in all participants.

**Figure 5:**
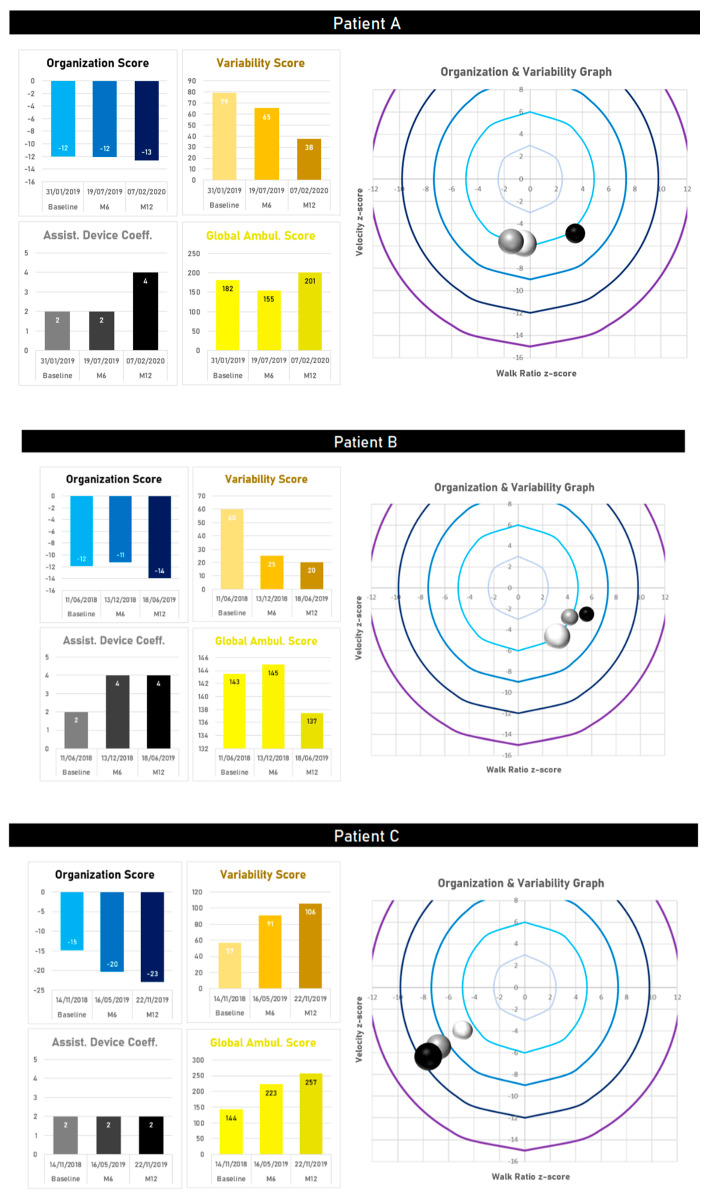
Follow-up over a period of six months for two individuals who switched from one cane to a rollator (patients **A** and **B**) and for an individual walking with a cane (patient **C**). Time progression is illustrated by the transition from lighter to darker colors.

In the three participants walking with a cane at baseline, two (A and B) transitioned to a rollator, while the third continued to utilize a cane (C). Patient A (Figure 5) transitioned to a rollator twelve months after baseline. During the first six months, this participant was still using a cane. The walking speed was reduced at minus six z-scores, but the walk ratio was within the control range, leading to OrgS of 12. Gait variability was exceedingly high (VarS > 65). Adoption of a rollator slightly attenuated the lower gait speed (minus five z-scores). However, Patient A’s gait pattern changed in favor of step length over cadence, and the level of variability decreased by half. Patient B (Figure 5) used a cane at baseline, with a low speed and high contribution of step length over cadence. After transitioning to a rollator six months after baseline, gait variability decreased by more than 50%, while keeping OrgS at the same level. After six months with the rollator, the level of gait variability was even better, and speed was maintained, however, this was achieved by a longer step length and unchanged cadence. Overall, although the use of a rollator increased the assistive device coefficient (two for a cane to four for a rollator), Patient B’s Global Ambulation Score improved from 143 at baseline to 137 one year later. Patient C (Figure 5), who still ambulated with a cane one year after baseline, demonstrated a valuable evolution to approaching longitudinal gait deterioration and identification of when to transition from cane to rollator. Gait speed at baseline was low (minus four z-scores), with a contribution of the cadence higher than step length (minus five z-score for the walk ratio), and a VarS at 57. Over twelve months of follow-up, a decrease in speed was identified, secondary to decreased step length with a constant cadence. This led to a worsening of the OrgS (−15 at baseline, −20 at six months, −23 at twelve months), while increasing VarS by 46% (57 at baseline, 91 at six months and 106 at twelve months). There was also a significant increase in GAS from 144 at baseline to 257 after one year.

## 4. Discussion

A new scoring system inspired by motor control theories has been made for gait parameter analysis. We assembled the background and computations using the theoretical and methodological sections of this paper, followed by extensive data analysis. The participants’ gait data were primordial in comparing theory to clinical reality and highlight the value of these new outcome measures.

The previous comparison between spatiotemporal parameters in individuals with FRDA and healthy controls during preferential ambulation demonstrated a range of alterations in relation to the disease: reduced ambulation speed, cadence, and swing phase, shortened step and stride length, increased base of support, step and stride time, stance phase, double support phase, and a larger amount of variability [3,19,52,53,54,55]. There is an indisputable trend, due to the neurodegenerative nature of FRDA, evolving towards an inability to walk, usually 10 to 20 years after the first symptoms [15], and a good relationship between gait parameters and the clinical status assessed by scales or functional tests has been reported [19,52,53,55]. However, although the most common of hereditary ataxias, FRDA remains a rare disorder and researchers are often faced with data from a small sample size that yield low statistical power. When FRDA progresses and gait worsens, assistive devices such as canes, crutches, walkers, or rollators become necessary to facilitate safe, upright locomotor function, maintain mobility, and prevent the detrimental effects of immobility [19]. From this perspective, in a small sample, it is difficult to ignore the statistical weight that the gait data may have captured in individuals using the most supportive device compared to independent ambulators, not only because individuals using a rollator would demonstrate the most affected parameters but also because their ambulation pattern represents a different type of locomotion when compared to those ambulating independently.

Our results, based on the characterization of two components, organization and variability, provide a less categorical, more detailed picture of gait characteristics in FRDA. First, in accordance with the hypothesis which led to this representation of gait, individuals with FRDA demonstrate various organization patterns and the same value for a parameter (e.g., gait speed) does not imply an equivalent organization nor interpretation. The use of the walk ratio offers complementary information to represent the gait. This was illustrated, for example, by the three individuals with FRDA who ambulated with a cane (see Figure 3, grey bubbles). They all presented about the same speed but three different walk ratios: one favoring cadence, one favoring step length, and one displaying a normal contribution of cadence and step length. Similarly, we outlined the requirement of concurrent consideration of the variability component of gait, the same organization possibly leading to different levels of variability. Contrary to common belief, where more alteration in the gait pattern (its organization) is thought to lead to more variability, the Variability Scores were higher in some individuals ambulating independently than others with a major deterioration of the Organization Score and who needed a rollator to ambulate.

The examination of the Organization Score and the Variability Score in relation to the clinical score, the SARA gait score (SARA-GS), instead supported the dynamic systems theory. A system will tend to stay in an organization until perturbations overpass the capacity of regulation. If residual capacities of adaptation allow adequate regulation to answer the perturbation or the adoption of a biomechanically safer, less destabilizing gait pattern, an individual would likely be able to remain ambulating without aids.

The clinical features of FRDA and their effects on gait do not follow the same pathway in all patients. A specific organization does not warrant that two patients will have the same level of stability, with each one having their constraints. The various Organization Scores highlight these inter-individual differences, which are not systematically reflected otherwise by clinical assessment. For example, a patient with triceps retractation and equinus feet who walks by mostly falling forward and another one with relatively preserved proprioception, slow walking but correct foot roll-over, could be both classified with the SARA-GS as a stage 2, which is defined as a gait “clearly abnormal, tandem walking >10 steps not possible”. On the other hand, the SARA-GS stages better reflect the increasing instability and related variability of patients. For example, stage 4 considers that intermittent support of a wall is required not to fall. Consequently, we did not see changes in the Organization Score in relation to the SARA-GS among individuals ambulating independently or those using a rollator, everyone having their constraints and organization of gait. Nevertheless, a longitudinal increase was seen in the Variability Score as the requirement of an assistive device became evident.

The transfer to ambulating with an assistive device usually brings more stability. Therefore, we have seen lower variability in the first stages involving the requirement of a supportive device (e.g., stages 5–6 of the SARA-GS) than in previous stages of independent ambulation. On the other side, the use of an ambulation device, especially a rollator, further limits the possible organizations (i.e., less variability of organizations among the individuals using a rollator), and we observed a trend towards decreasing gait velocity and the role of the cadence in velocity (i.e., increased walk ratio). Therefore, the Organization Score longitudinally worsens from independent ambulators to individuals with a rollator. Finally, while the global value given by the Global Ambulation Score took into consideration the Organization Score, the Variability Score, and the level of an assistive device, it demonstrated a logarithmic relationship with the SARA-GS, implying that it might be a valuable outcome measure for longitudinal disease progression.

The method that we present displays promise for understanding the gait in FRDA patients and warrants future research to complete these preliminary results. All patients do not follow the same pathway from initial gait troubles to the total loss of ambulation: some use a cane before switching to a rollator, while others need to transition from independent ambulation to the use of a rollator. Again, based on an individual’s constraints, the transition towards a supported gait can be abrupt or gradual. One of the most interesting features requiring further investigation could be the identification of concurrent levels of organization and variability, which are difficult to sustain and could indicate the inevitable requirement of an assistive device.

As described in the methods, coefficients have been introduced in the Global Ambulation Score to reflect the increase in support provided by the assistive device, from the unilateral support provided by a cane to bilateral support provided by the rollator. Coefficients for walking aids one, two, and four were arbitrarily selected for unassisted walk, use of a cane, and use of a rollator, respectively. While some other factors may have been possible, the Organization and Variability Scores are both mathematically independent of the coefficient for walking aids. On the other hand, assigning coefficients according to the type of assistive device raises the question about the effectiveness of the method and the potential for distortive effects on longitudinal follow-up when current clinical tools solely evaluate performance by time to walk a certain distance.

Regarding the normative dataset used to derive the scores from gait parameters recorded in the control population, an adjustment would be required for patients younger than the youngest subject included in our study (i.e., 11 years old). Spatiotemporal gait parameters mostly evolve before the age of seven. Following this age, changes are mostly attributed to growth, and our methodology already considers normalized parameters. However, the gait variability still decreases after the age of seven, mainly until 11 years of age, reflecting the neuromaturation of dynamic balance abilities [56,57]. Normative data for younger children are already available and could be easily implemented.

In this pilot study, we focused the methodology and the results on a small sample size of individuals with Friedreich’s ataxia, however, further study with a larger sample size is warranted to thoroughly assess the properties of the proposed metrics. The concept is not limited to this population and might be extended to other populations, such as older adults, or other paradigms, such as the effect of dual-tasking. As we mentioned before, adopting strategies like stiffening is likely to be beneficial in avoiding a loss of balance during simple postural and walking tasks. However, as suggested by Young and Williams [58], these changes “will ultimately compromise performance in dynamic and highly demanding functional tasks”.

## Figures and Tables

**Table 1:** Demographics, clinical information, and gait parameters for the persons living with Friedreich’s ataxia (FRDA).

	Unassisted		Cane		Rollator	
	Mean (SD)	CI_95%_	Mean (SD)	(Min; Max)	Mean (SD)	CI_95%_
n	9		3		7	
Female sex (%)	55.6		66.7		14.3	
Age (years)	14.3 (3.2)	[11.9, 16.8]	29.3 (17.1)	[18.1, 49.1]	34.0 (16.8) *	[18.4, 49.5]
Body Mass Index (kg/m²)	20.6 (3.4)	[18.0, 23.2]	25.0 (9.7)	[18.3, 36.1]	24.6 (4.2)	[19.3, 30.5]
Age at FRDA onset (years)	7.1 (3.2)	[4.7, 9.6]	15.7 (10.0)	[8.0, 27.0]	16.1 (8.9)	[7.9, 24.4]
Time since onset (years)	7.2 (3.1)	[4.9, 9.6]	13.7 (8.0)	[6.1, 22.1]	17.8 (8.7) *	[9.8, 25.9]
Repeat Length GAA1	691 (173)	[547, 836]	661 (472)	[223, 1160]	515 (221)	[311, 720]
Repeat Length GAA2	947 (172)	[803, 1091]	937 (421)	[452, 1200]	900 (91)	[815, 984]
SARA Gait Score	3.0 (0.7)	[3, 4]	4.7 (0.6)	[4, 5]	6.1 (0.7) *	[5, 7]
SARA Stance	2.0 (0.9)	[1, 3]	2.7 (0.6)	[2, 3]	2.3 (1.4)	[1, 4]
Functional Staging for Ataxia Score	2.3 (0.7)	[2, 3]	3.0 (0.0)	[3, 3]	4.0 (0.5) *	[3, 5]
FARS Total Upright Stability Score	20.4 (5.1)	[16, 24]	24.1 (2.1)	[22, 26]	23.9 (4.3)	[20, 28]
Modified FARS	34.7 (10.8)	[26, 43]	43.5 (2.9)	[41, 47]	39.9 (6.5)	[34, 46]
Mean of Normalized Velocity (Vn _mean_)	0.28 (0.05)	[0.24, 0.32]	0.18 (0.03)	[0.15, 0.21]	0.12 (0.05) *	[0.08, 0.16]
Mean of the Normalized Walk ratio (WR _mean_)	0.49 (0.08)	[0.43, 0.55]	0.46 (0.15)	[0.31, 0.60]	0.71 (0.13) *	[0.59, 0.83]
Coeff. of variation for Normalized Velocity (Vn _CV_)	8.8 (4.3)	[5.5, 12.1]	11.9 (5.1)	[6.5, 16.5]	12.8 (6.1)	[7.2, 18.4]
Coeff. of variation for Normalized Walk Ratio (WR _CV_)	10.3 (4.3)	[7.0, 13.6]	21.9 (4.3)	[17.2, 25.4]	11.8 (2.2)	[9.8, 13.8]
Organization Score	−5.1 (4.9)	[−8.9, −1.4]	−12.9 (1.6)	[−14.8, −11.9]	−20.7 (5.8) *	[−26.0, −15.3]
Organization Score (absolute value)	6.1 (3.3)	[3.6, 8.7]	12.9 (1.6)	[14.8, 11.9]	20.7 (5.8) *	[15.3, 26.0]
Variability Score	35.7 (15.8)	[23.6, 47.9]	65.3 (11.9)	[57.1, 78.9]	50.5 (23.4)	[28.9, 72.1]
Global Ambulation Score	41.9 (17.5)	[28.5, 55.3]	156.3 (22.0)	[143.5, 181.8]	284.8 (106.5) *	[186.3, 383.3]

* significant difference (*p* < 0.05) between the unassisted group and the rollator group; cane group was only composed by three persons. FRDA: Friedreich’s ataxia; SARA: Scale for Assessment and Rating of Ataxia; FARS: Friedreich’s Ataxia Rating Scale.

## Data Availability

The data presented in this study are available on request from the corresponding author. The data are not publicly available due to privacy.
